# Modulation of the gut microbiome and Firmicutes phylum reduction by a nutraceutical blend in the obesity mouse model and overweight humans: A double‐blind clinical trial

**DOI:** 10.1002/fsn3.3927

**Published:** 2024-01-19

**Authors:** Victor Nehmi‐Filho, Jessica Alves de Freitas, Lucas Augusto Franco, Roberta Cristina Martins, José Antônio Orellana Turri, Aline Boveto Santamarina, Joyce Vanessa da Silva Fonseca, Ester Cerdeira Sabino, Bruna Carvalho Moraes, Erica Souza, Gilson Masahiro Murata, Silvia Figueiredo Costa, Paulo Sérgio Alcântara, José Pinhata Otoch, Ana Flávia Marçal Pessoa

**Affiliations:** ^1^ Laboratório de Investigação Médica (LIM‐26), Laboratório de Produtos e Derivados Naturais, Departamento de Cirurgia Universidade de São Paulo Faculdade de Medicina Pacaembu Brazil; ^2^ Departamento de Doenças Infecciosas e Parasitárias, Laboratório de Parasitologia Médica (LIM‐46) Universidade de São Paulo Instituto de Medicina Tropical de São Paulo Jardim America Brazil; ^3^ Departamento de Ginecologia e Obstetrícia, Grupo de Pesquisa em Economia da Saúde Universidade de São Paulo Faculdade de Medicina Pacaembu Brazil; ^4^ Departamento de Doenças Infecciosas e Parasitárias, Laboratório de Investigação Médica em Protozoologia, Bacteriologia e Resistência Antimicrobiana (LIM‐49) Universidade de São Paulo Instituto de Medicina Tropical de São Paulo Jardim America Brazil; ^5^ Laboratório de Investigação Médica (LIM‐31), Laboratório Investigação Médica em Patogênese e Terapia dirigida em Onco‐Imuno‐Hematologia Universidade de São Paulo Faculdade de Medicina, Universidade de São Paulo Hospital das Clínicas Cerqueira César Brazil; ^6^ Monte Azul Ambulatório Jardim Mirante Brazil; ^7^ Laboratório de Investigação Médica (LIM‐29), Laboratório de Nefrologia Celular, Genética e Molecular, Departamento de Clínica Médica Universidade de São Paulo Faculdade de Medicina Pacaembu Brazil; ^8^ Departamento de Cirurgia Universidade de São Paulo Hospital Universitário de São Paulo Butantã Brazil; ^9^ Efeom Nutrition Universidade de São Paulo Faculdade de Medicina Pacaembu Brazil

**Keywords:** gut microbiota, overweight, prebiotics, silymarin

## Abstract

Overweight and obesity are closely linked to gut dysbiosis/dysmetabolism and disrupted De‐Ritis ratio [aspartate aminotransferase (AST)/alanine aminotransferase (ALT) ratio], which may contribute to chronic noncommunicable diseases onset. Concurrently, extensive research explores nutraceuticals, and health‐enhancing supplements, for disease prevention or treatment. Thus, sedentary overweight volunteers were double‐blind randomized into two groups: Novel Nutraceutical_(S) (without silymarin) and Novel Nutraceutical (with silymarin). Experimental formulations were orally administered twice daily over 180 consecutive days. We evaluated fecal gut microbiota, based on partial 16S rRNA sequences, biochemistry and endocrine markers, steatosis biomarker (AST/ALT ratio), and anthropometric parameters. Post‐supplementation, only the Novel Nutraceutical group reduced *Clostridium clostridioforme* (Firmicutes), Firmicutes/Bacteroidetes ratio (F/B ratio), and De‐Ritis ratio, while elevating *Bacteroides caccae* and *Bacteroides uniformis* (Bacteroidetes) in Brazilian sedentary overweight volunteers after 180 days. In summary, the results presented here allow us to suggest the gut microbiota as the action mechanism of the Novel Nutraceutical promoting metabolic hepatic recovery in obesity/overweight non‐drug interventions.

## Graphical abstract

The nutraceutical composition containing silymarin reduces the Firmicutes/Bacteroidetes ratio (F/B ratio). The nutraceutical composition of silymarin also lowers the De‐Ritis ratio, a biomarker for liver complications. The nutraceutical composition enriched with silymarin promotes an increase in *Bacteroides caccae* and *Bacteroides uniformis*, both belonging to the Bacteroidetes phylum.

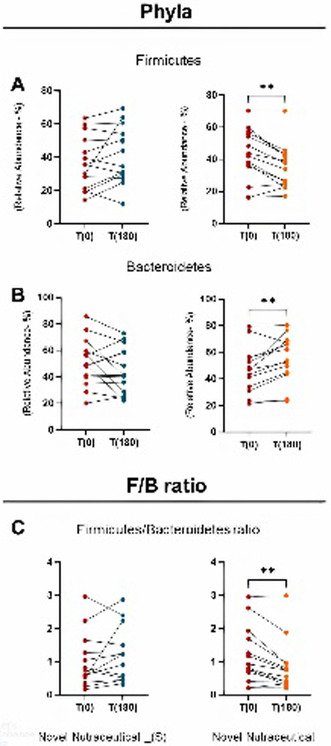

## 1 INTRODUCTION

Gut dysbiosis/dysmetabolism (Dao et al., 2016; Fedor & Kelley, 2013; Garcia‐Gutierrez & Sayavedra, 2022; Maloy & Powrie, 2011) and meta‐inflammation are associated with overweight/obesity, extending beyond genetics and the socioeconomic factors (Nehmi‐Filho, Santamarina, et al., 2023). According to projections, the number of adults affected by obesity is expected to surpass 1.3 billion by 2030 (Estivaleti et al., 2022; Haththotuwa et al., 2020). In Brazil, the prevalence of overweight in adults was 40.5% in 2020 (Kodaira et al., 2021).

The overweight/obese state influences metabolic dysfunction (Lassale et al., 2018) and is associated with AST/ALT ratio (AST)/alanine aminotransferase (ALT) ratio in coronary/arterial heart diseases (Liu & Liu, 2022), nonalcoholic liver disease (NAFLD) (Harrison et al., 2008), and alterations in the microbiome composition (Dreyer & Liebl, 2018; Liu et al., 2021).

A healthy human gut microbiota contains over 9 million bacteria characterized by a higher bacterial diversity and the *Bacteroidetes* and *Firmicutes phyla* (Dreyer & Liebl, 2018) representing 90% of the gut microbiota diversity and populations (Jayasinghe et al., 2016; León Aguilera et al., 2022; Rajani & Jia, 2018). Increased Firmicutes, and decreased Bacteroidetes bacteria (León Aguilera et al., 2022), as well as elevated Firmicutes/Bacteroidetes ratio (F/B) (Houtman et al., 2022) and *Blautia*/*Bacteroides* ratio augmentation (Kim et al., 2022), are intrinsically linked with physiological processes and body composition in obesity.

*Our research* finds that a novel nutraceutical composition can be used as a strategy for preventing or improving inflammatory and metabolic ailments (Nehmi et al., 2021; Nehmi‐Filho, Santamarina, et al., 2023; Santamarina et al., ), besides decreased de F/B ratio in gut microbiota (Nehmi‐Filho, de Freitas, et al., 2023). Apart from prebiotics, medicinal plants could serve as sources for the gut microbiota to produce secondary compound products, such as short‐chain fatty acids, postbiotics, and other bioactive molecules, that help with oxidative stress defense and promote favorable metabolic and immunological conditions not only in the gut environment but also systemically (Nehmi‐Filho, de Freitas, et al., 2023; Vamanu, 2019).

The assessment of the specific impacts of minerals (Skrypnik & Suliburska, 2018), prebiotics (He & Shi, 2017; Santos‐Marcos et al., 2019), and *Silybum marianum* (Milk thistle) seeds. *Silybum marianum* seeds contain silymarin, a flavonolignan mixture comprising four isomers: silybin, isosilybin, silydianin, and silychristin (Kumar et al., 2020; Shen et al., 2019; Xu et al., 2022), often administered in high concentrations (Nehmi‐Filho, de Freitas, et al., 2023).

Here, we study the gut microbiota composition from sedentary overweight volunteers 180 days post‐supplementation with two different compositions of supplements [Novel Nutraceutical_(S) and Novel Nutraceutical] made by a blend of β‐glucan, prebiotics, minerals, and *Silybum marianum* or milk thistle (silymarin seed extract) in low concentrations. The primary proposition is that the supplements can modulate the gut microbiota providing an improvement in hepatic and anthropometric parameters without changing diet and exercise patterns.

## 2 ETHICAL CONSIDERATIONS AND METHODOLOGY

### 2.1 Ethics committee approvals

All experimental procedures conducted in this study received prior approval from the respective local research ethics committees of the University of São Paulo (São Paulo, Brazil). The study adheres to international guidelines and standards.

### 2.2 Human protocol

#### 2.2.1 Enrollment of participants and experimental design

**FIGURE 1:**
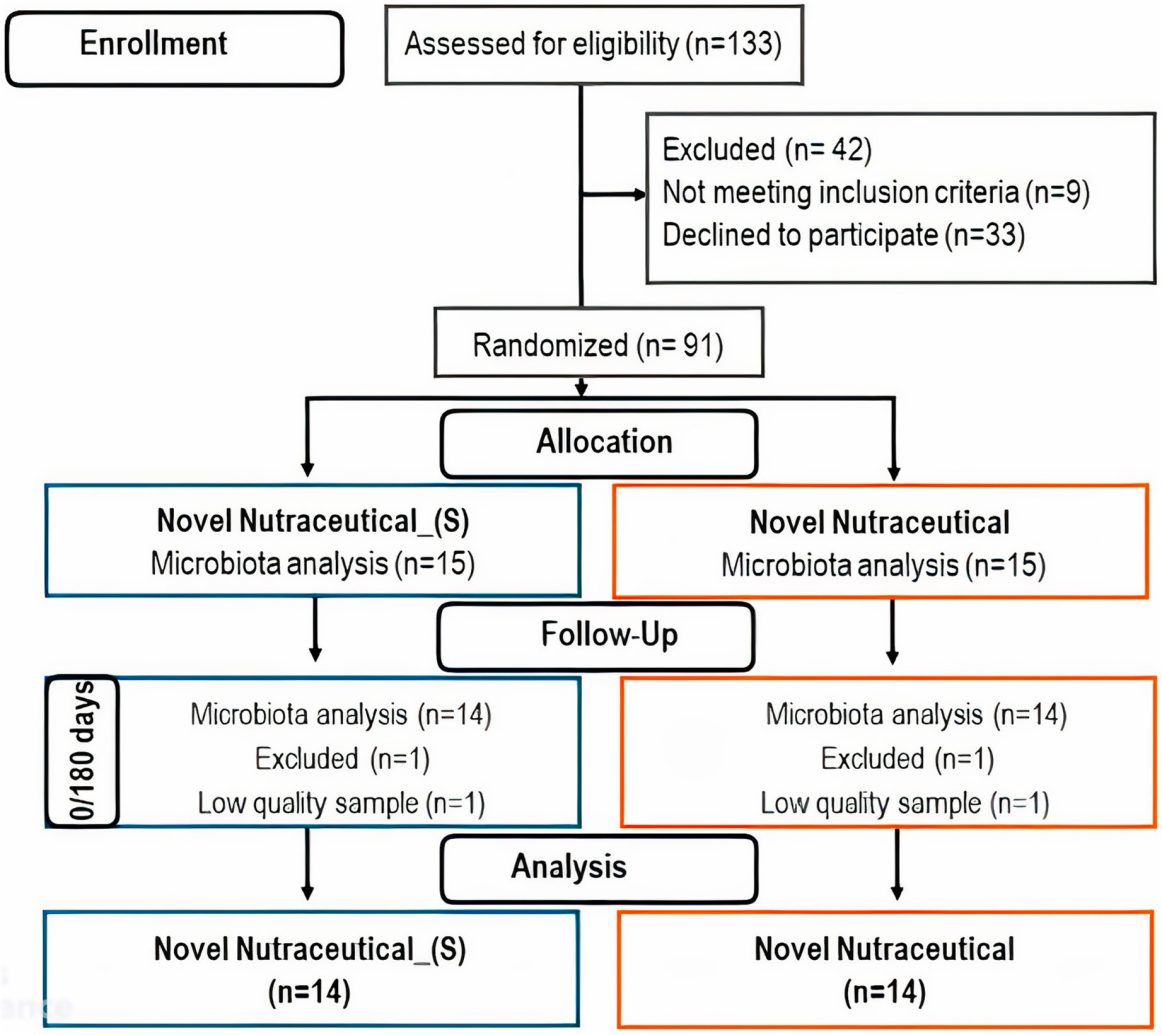
Consolidated Standards of Reporting Trials (CONSORT) flowchart describing the recruitment of volunteers and the experimental design carried out on this clinical trial.

The “Nutraceutical Supplement Trial” enrolled 133 sedentary volunteers from March to June 2021, utilizing online advertising and the *Ambulatório Monte Azul* (São Paulo, Brazil). This double‐blind, randomized trial assessed volunteers at baseline (T0) and after 180 days of supplementation (T180). Inclusion/exclusion criteria, sample size calculations, and supplement guidelines followed previously described protocols (Nehmi‐Filho, Santamarina, et al., 2023). The CONSORT guidelines were employed, and the trial flow is detailed in Figure 1. A total of 133 volunteers responded to the call, with 33 withdrawing and 9 not meeting the inclusion criteria. Randomization resulted in two groups: Novel Nutraceutical Supplement_(S) (*n* = 15) and Novel Nutraceutical (*n* = 15), with 14 participants in each group completing the study. Blood and feces samples were collected at T0 and T180 (Table 1).

**TABLE 1:** Population's descriptive characteristics, anthropometrics, and serum data.

Variables	Novel Nutraceutical_(s)		Novel Nutraceutical		*p*
	T0	T180	T0	T180	
Sample size	14		14		
Gender (female/male)	8/6		10/4		
Age (years)	54.14 ± 5.65		56.14 ± 4.49		–
Anthropometrics					
Height (cm)	164.4 ± 10.97		161.1 ± 7.90		–
Body mass (kg)	72.52 ± 10.89	72.29 ± 10.46	–	71.25 ± 13.13	–
BMI (kg/m^2^)	26.79 ± 2.86	26.71 ± 2.82	–	27.44 ± 3.68	–
Neck (cm)	36.61 ± 2.98	36.1 ± 2.86	–	35.5 ± 3.47	–
WC‐mid (cm)	90.24 ± 7.39	88.96 ± 8.18	–	86.97 ± 11.71	–
Hip (cm)	102.8 ± 4.38	103.4 ± 5.93	–	102.9 ± 7.12	–
WC‐IC (cm)	97.14 ± 5.91	98.29 ± 7.12	–	97.21 ± 9.08	–
WHtR	0.55 ± 0.05	0.55 ± 0.06	–	0.55 ± 0.09	–
WHR	0.88 ± 0.06	0.86 ± 0.07	0.88 ± 0.08	0.84 ± 0.10	**.043** ^b^
Hormones and biochemistry exams					
AST/ALT ratio	2.47 ± 1.14	1.47 ± 0.37	2.62 ± 1.18	2.00 ± 0.82	**.006** ^a^
Cortisol (μg/dL)	14.65 ± 3.34	11.61 ± 5.08	14.19 ± 4.21	12.71 ± 4.96	–
TSH (mUI/L)	4.11 ± 2.54	2.61 ± 1.21	2.86 ± 1.72	4.20 ± 4.71	**.011** ^a^

*Note*: Data values are expressed as mean ± SEM.

Abbreviations: AST/ALT ratio (Aspartate aminotransferase (AST)/Alanine aminotransferase (ALT) ratio); BMI, body mass index; T0, day zero; T180, 180 days post‐supplementation; TSH, thyroid‐stimulating hormone; WC‐mid, waist circumference in middle abdomen; WC‐IC, waist circumference in Iliac Crest; WHtR, waist‐to‐height ratio; WHR, waist‐to‐hip ratio.

^a^ Significance difference Novel Nutraceutical_(S)T0 versus T180.

^b^ Significance difference Novel Nutraceutical T0 versus T180.

#### 2.2.2 Nutraceutical composition formulas

Two different formulations were tested herein (Patent number BR 102020 016156 3) as previously published by Nehmi‐Filho, Santamarina, et al. (2023) as described below. Novel Nutraceutical _(S) (*n* = 14): zinc (Zn) 1%, magnesium (Mg) 1% (Purifarma Distribuidora Química e Farmacêutica, São Paulo, Brazil), Fructooligosaccharide (FOS) 45% (NutraFlora®, Westchester, Illinois, USA), selenomethionine (Se) 0.01%, Galactooligosaccharide (GOS) 10%, tixosil 5%, and 1.3/1.6‐(β‐glycosidic bonds) yeast β‐glucans (*Saccharomyces cerevisiae*) 6% (Biorigin, São Paulo, Brazil); and Novel Nutraceutical (*n* = 14) contained the following components: zinc (Zn) 1%, magnesium (Mg) 1% (Purifarma Distribuidora Química e Farmacêutica, São Paulo, Brazil), Fructooligosaccharide (FOSs) 45% (NutraFlora®, Westchester, Illinois, USA), selenomethionine (Se) 0.01%, Galactooligosaccharide (GOS) 10%, tixosil 5%, 1.3/1.6‐(β‐glycosidic bonds) yeast β‐glucans (*Saccharomyces cerevisiae*) 6% (Biorigin, São Paulo, Brazil), and *Silybum marianum* (3.11% of seed extract) (SM Empreendimento Farmacêutica LTDA, São Paulo, Brazil). The formulations adhered to EFSA (European Food Safety Authority (EFSA), 2017), recommendations, were prepared by *Solis Magistral Farmácia Homeopática Sensitiva* (São Paulo, Brazil), ensuring the trial blind spot.

#### 2.2.3 Body measurements of the volunteer

Body measure, including weight, height, hip, waist, neck circumferences, Body Mass Index (BMI) (BMI = body mass(kg)/height(m)^2^), using Body Composition Scale 2 (Xiaomi Mi, Beijing, China), Waist‐to‐Height Ratio (WHtR), and Waist‐to‐Hip Ratio (WHR), were recorded at T0 and T180 using standardized methods.

#### 2.2.4 Dietary intake data and International Physical Activity Questionnaire (IPAQ)

Participants' dietary intake data were obtained from a 3‐day food diary and analyzed using DietPro software (version 6.1). Physical activity was assessed through the International Physical Activity Questionnaire (IPAQ), categorizing activities based on intensity (Matsudo et al., 2001).

#### 2.2.5 Aspartate aminotransferase (AST)/ alanine aminotransferase (ALT) ratio (De‐Ritis), and endocrines parameter

Blood samples, collected between 7:00 a.m. and 9:00 a.m., were analyzed for (AST) (U/L), (ALT) (U/L), cortisol (μg/dL), and thyroid‐stimulating hormone (TSH) (mUI/L). The De‐Ritis ratio was obtained, using concentration in serum samples, according to Rief et al., 2016. Analyses were performed by “*Fleury Medicina e Saúde*” laboratory.

#### 2.2.6 Firmicutes/Bacteroidetes (F/B) and *Blautia/Bacteroides* ratios

The F/B ratio was calculated by dividing the relative abundances of Firmicutes by the relative abundance of the Bacteroidetes (Houtman et al., 2022). The *Blautia/Bacteroides* ratio was calculated by dividing the relative abundances of *Blautia* by the relative abundance of *Bacteroides* (Kim et al., 2022), providing insights into the microbial composition.

### 2.3 Mice protocols

#### 2.3.1 Supplement compositions

The supplement formulations, Novel Nutraceutical_(S) and Novel Nutraceutical (patent number: BR 102020 016.156 3), were developed and tested by our group, as previously described by Nehmi et al. (2021) and Santamarina et al. (2022). The dosage was adapted for mice based on the Animal Equivalent Dose (AED) equation (Nair & Jacob, 2016). The composition was administered in a 2% carboxymethylcellulose solution.

#### 2.3.2 Animal experimentation

**FIGURE 2:**
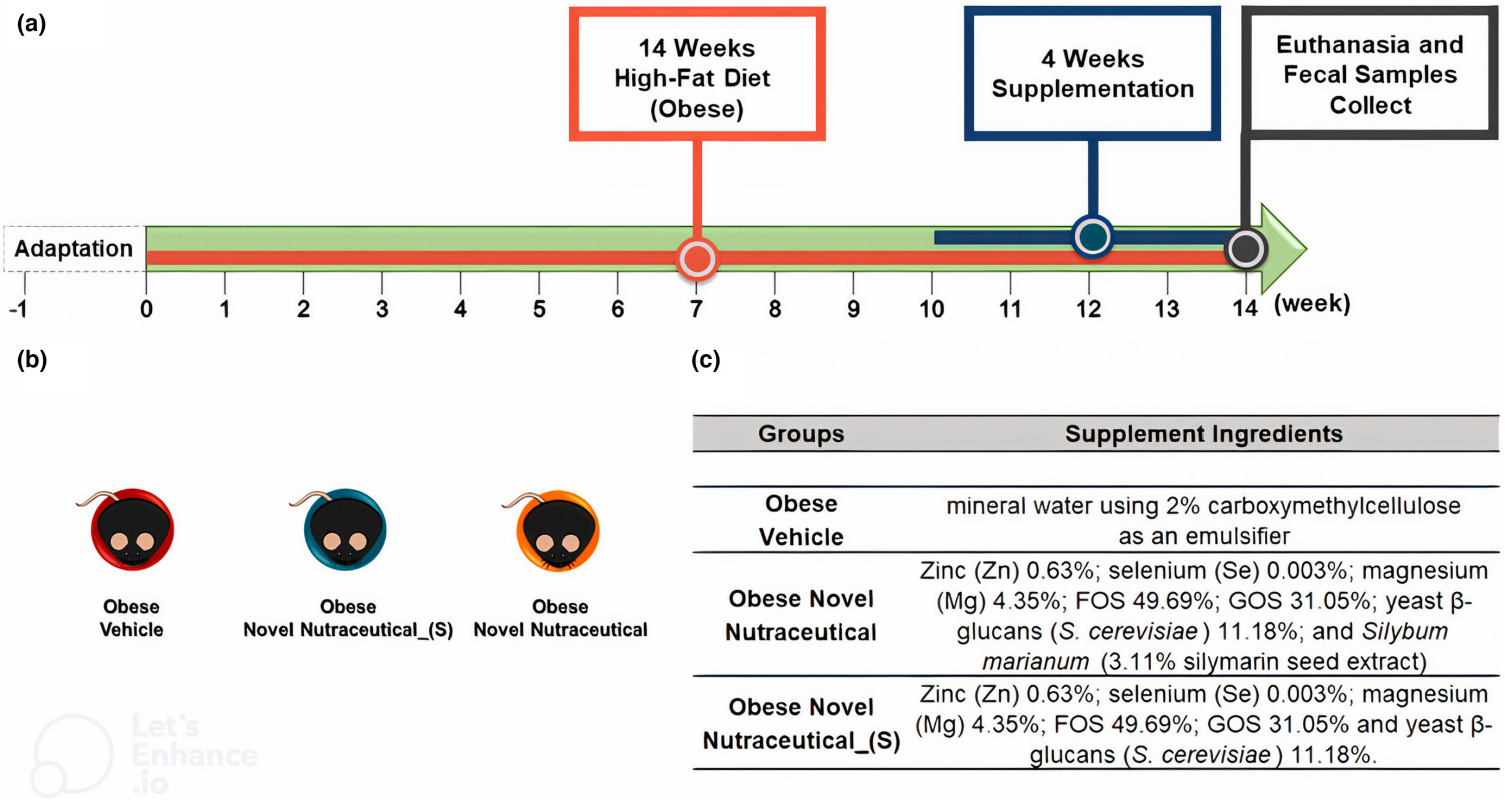
Adapted figure (Nehmi‐Filho, de Freitas, et al., 2023). Schematic outline of the experimental procedure, supplementation time, and supplement compositions common to all animals. (a) Timeline, (b) experimental groups, and (c) specific supplement composition described by experimental group.

Adult C57BL/6N male 60‐day‐old mice were acclimated at the vivarium in controlled conditions of temperature (24 ± 2°) and a 12‐h light/dark cycle. For the development of obesity, we fed mice a commercial high‐fat diet protocol (5.25 kcal/g, 30% saturated fat (mainly lard), 35.95% carbohydrates, and 20% proteins) (Moreira et al., 2018) (Prag Soluções Biociências, Jau, Sao Paulo, Brazil). Animals were fed ad libitum for 14 weeks. In the 10th week, the mice were divided into experimental groups receiving oral supplementation by gavage for 28 days. Animals were submitted to the following supplementations: Obese Vehicle (*n* = 7) – 2% carboxymethylcellulose; Obese Novel Nutraceutical_(S) (*n* = 5) – zinc (Zn) 0.63%, selenium (Se) 0.003%, magnesium (Mg) 4.35%, Fructooligosaccharide (FOS) 49.69%, Galactooligosaccharide (GOS) 31.05%, and 1,3/1,6‐(β‐glycosidic bonds) β‐glucans from yeast (*Saccharomyces cerevisiae)* 11.18% (Yes Synergy, Campinas, São Paulo, Brazil); Obese Novel Nutraceutical (*n* = 7) – zinc (Zn) 0.63%, selenium (Se) 0.003%, magnesium (Mg) 4.35%, Fructooligosaccharide (FOS) 49.69%, Galactooligosaccharide (GOS) 31.05%, 1,3/1,6‐(β‐glycosidic bonds) β‐glucans from yeast (*Saccharomyces cerevisiae*) 11.18% (Yes Synergy, Campinas, Sao Paulo, Brazil), and silymarin extract (*Silybum marianum*) 3.11% (Ningbo Vitax Biotech Co., China) (Figure 2). Also, Nehmi et al. (2021) and Santamarina et al. (2022) previously described the animal protocol biochemistry, anthropometric parameters, and diet composition in detail.

#### 2.3.3 Microbiome analysis

##### Sample collection in mice

Stool samples were collected from the gut colon within a controlled environment to prevent contamination. Samples were carefully stored in sterile 2 mL tubes at a temperature of −80°C until the DNA extraction.

##### Sample collection in human

Each participant collected around 1 g of feces. The sample was submerged in guanidine stool preservation medium for microbiome immediately after collection and kept with temperature control, 2–8°C for sample transport and 0 to ‒80°C for storage (Ribeiro et al., 2018).

##### Genomic extraction

Genomic material was acquired through DNA extraction, employing approximately 0.25 g of feces and utilizing the DNeasy PowerSoil Kit (Qiagen, Germantown, MD, USA). The extracted material was subsequently preserved at −20°C until the library preparation stage.

##### Library preparation and sequencing

The library preparation and sequencing procedures are extensively detailed in Nehmi‐Filho, de Freitas, et al. (2023). In summary, for prokaryotic community analysis, 16S rRNA (V4 region) sequences were directly amplified and sequenced using 515F/806R. This amplification was carried out using a bacterial/archaeal primer set, specifically 515F/806R (Caporaso et al., 2011). The sequencing process was performed according to the manufacturer's instructions (Thermo Fisher Scientific, Waltham, MA, USA) using the Ion Chef System and the Ion S5 platform.

##### Bioinformatic analysis

The detailed bioinformatic analysis steps can be found in Nehmi‐Filho, de Freitas, et al. (2023). In summary, the 16S rRNA gene data underwent preprocessing and diversity estimation using *Quantitative Insights Into Microbial Ecology* (QIIME 2) version 2020.11 (Bolyen et al., 2019). The average number of sequences per sample in mouse analysis was 66,922, and in the human analysis was 40,617. The data were denoised with DADA2 (via q2‐dada2) using default parameters, which included a length threshold of 200 bp and an average quality Phred score of ≥30. This denoising step generated amplicon sequence variants (ASVs) (Callahan et al., 2016). The mouse analysis identified 1286 ASVs; and the human analysis identified 2433 ASVs. Following the construction of a phylogenetic tree, alpha and beta diversity metrics were calculated using Q2‐diversity. The human samples were rarefied to 19,639 and mouse samples to 38,122 sequences per sample (Faith, 1992) before estimating these metrics.

The taxonomic classification of ASVs was performed using the Q2‐feature classifier (Bokulich et al., 2018), specifically employing the naive Bayes classifier against the Greengenes 13_8 99% OTUs (Operational Taxonomic Unit) reference sequences (Mandal et al., 2015). The composition of microbiota communities was summarized at various taxonomy levels, including species, genera, families, orders, classes, and phyla ranks.

Additionally, after the samples were rarefied to 19,639 sequences per sample, alpha diversity metrics such as Chao1, Simpson, OTUs, Pielou's evenness, Shannon diversity, and Faith's phylogenetic diversity were calculated. The beta diversity metrics employed were Jaccard distance, Bray–Curtis distance, and unweighted and weighted UniFrac distances.

To enhance the interpretability of microbiome data, we employed a heatmap visualization technique focusing on genera that exhibited differential representation between supplement groups. Differential abundance analysis was conducted utilizing the R package DESeq2, a robust tool designed for differential gene expression analysis based on the negative binomial distribution (version 4.3.2). To pinpoint taxonomic characteristics contributing to variations between different study periods and supplement groups, we utilized the Linear Discriminant Analysis Effect Size (LEfSe) algorithm (version 1.1.2). LEfSe is a powerful method for identifying features that are statistically different with biological relevance. This analysis facilitated the determination of taxonomic signatures crucial for understanding microbiome variations associated with the experimental conditions.

### 2.4 Statistical analysis

Data were classified as parametric or nonparametric based on the Shapiro–Wilks and Smirnov–Kolmogorov test. Continuous parametric data were shown as mean ± standard deviation, and nonparametric as median and interquartile range. To compare the differences in the unpaired groups, parametric (Student *t*‐test and ANOVA) or nonparametric (Mann–Whitney or Kruskal Wallis) tests were conducted when indicated, and categorical data were analyzed using chi‐square or Fisher's exact test. Univariate and multivariate logistic and linear regression were conducted to evaluate the main hormonal and anthropometric variables associated with using the Novel Nutraceutical Supplement presented as coefficients and 95%CI and RR and 95%CI. In addition, variables with *p* < .2 in the univariate regression were posteriorly analyzed in the multi‐variable regression. For all analyses, significance was determined as *p* < .05. Comparisons between groups involved were made by paired t‐test. Analyses were performed using STATA® 14.0 (Stata Corp. LCC, College Station, TX, USA) and GraphPad Prism 9.0 (GraphPad Software, La Jolla, CA, USA) software. The genera that were differentially represented between supplement groups were determined using the R package DESeq2 – Differential gene expression analysis based on the negative binomial distribution – (4.3.2). To determine the taxonomic characteristics most likely to explain differences between periods and supplement groups, we employed the algorithm Linear discriminant analysis Effect Size (LefSe 1.1.2) (Nearing et al., 2022).

## 3 RESULTS

### 3.1 Novel Nutraceutical compositions reduce AST/ALT ratio and WHR independent of diet and physical activity influences

We investigated the impact of two distinct supplements, each comprising seven elements, on specific health indicators without altering diet or physical activity. These supplements include a mineral blend containing zinc, selenium, and magnesium, prebiotics such as FOS and GOS, yeast‐β glucan, and silymarin extract (*Silybum marianum*). The key distinction between the supplements lies in the presence of silymarin extract (*Silybum marianum*), renowned in herbal medicine for the potential to reduce fatty liver, fibrosis, and liver inflammation in both animals and humans (Gillessen & Schmidt, 2020; Kumar et al., 2020; Shen et al., 2019; Xu et al., 2022).

Beginning with the descriptive data of the samples, it is evident that both groups had identical sample sizes, without dropouts during the supplementation, indicating high adherence and acceptance of the supplement among participants. The predominance of female participants aligns with studies showing a greater inclination among women to volunteer for research (Manteuffel et al., 2013; Otufowora et al., 2021). However, the sample remained homogeneous concerning age and height variables as presented in Table 1.

The BMI data in this study indicate that volunteers categorized as overweight were part of both groups from the start of the supplementation period. Analyzing the anthropometric data before and after supplementation revealed no variations in body mass parameters or measured circumferences between groups or over time. However, consumption of the Novel Nutraceutical led to a reduction in the WHR compared to initial values. This finding is significant, as a decreased WHR is associated with a lower risk of cardiovascular diseases. Additionally, examining blood hormones and biochemistry exams revealed noteworthy improvements in AST/ALT ratio and TSH levels following consumption of Novel Nutraceutical_(S), despite no changes in cortisol levels (Table 1).

Evaluation of dietary intake data through a 3‐day self‐report showed no modifications among the groups during the supplementation period in terms of energy (Kcal), protein, fiber, lipids, and cholesterol consumption. There was a slight increase in carbohydrate intake in the Novel Nutraceutical group at T180 compared to T0. This lack of substantial changes was expected, given that participants in this study were not subjected to dietary interventions and were instructed to maintain their eating habits throughout. This result reinforces the evidence that the positive outcomes observed are directly linked to supplement consumption and not influenced by changes in eating habits (Table 2).

**TABLE 2:** Populations' dietary intake data and physical activity levels are classified by the International Physical Activity Questionnaire (IPAQ).

Variables	Novel Nutraceutical_(S)		Novel Nutraceutical		*p*
	T0	T180	T0	T180	
Sample size	14		14		
Dietary intake					
Energy (Kcal)	1730 ± 436.8	1718 ± 413.2	1789 ± 300.4	1928 ± 516.1	–
Carbohydrates (g)	236.5 ± 52.51	225.4 ± 70.56	230.5 ± 45.7	269.3 ± 68.99	.018a^a^
Fiber (g)	12.77 ± 6.603	12.15 ± 5.357	12.83 ± 6.196	11.39 ± 5.188	–
Lipids (g)	53.71 ± 21.84	56.51 ± 19.88	60.71 ± 19.3	62.87 ± 30	–
Cholesterol (mg)	192.9 ± 97.39	201.0 ± 114.1	215.0 ± 99.65	294.7 ± 148.4	–
Proteins (g)	77.7 ± 30.32	79.37 ± 31.46	72.86 ± 18.31	74.74 ± 27.37	–
Physical activity level					
Sedentary	7.14% (*n* = 1)	14.28% (*n* = 2)	7.14% (*n* = 1)	21.43% (*n* = 3)	–
Irregularly active	42.86% (*n* = 6)	28.57% (*n* = 4)	78.57% (*n* = 11)	35.71% (*n* = 5)	–
Active	50% (*n* = 7)	57.14% (*n* = 8)	0% (*n* = 0)	35.71% (*n* = 5)	–
Very active	0% (*n* = 0)	0% (*n* = 0)	14.28% (*n* = 2)	7.14% (*n* = 1)	–

*Note*: Data values expressed as mean ± SEM.

^a^ Significance difference Novel Nutraceutical T0 versus T180.

Assessment of physical activity levels using the IPAQ, short version, among volunteers, revealed an increase in sedentary habits over time and a decrease in highly active volunteers. Moreover, the Novel Nutraceutical group primarily consisted of irregularly active volunteers. In contrast, the Novel Nutraceutical_(S) group comprised over 50% active volunteers but lacked participants classified as highly active. These findings suggest that neither group maintained a consistent practice of physical activity capable of influencing the results of this research through supplementation, as depicted in Table 2.

### 3.2 The Novel Nutraceutical supplement did not alter the diversity of gut microbiota but modulated the composition of phyla in overweight volunteers

**FIGURE 3:**
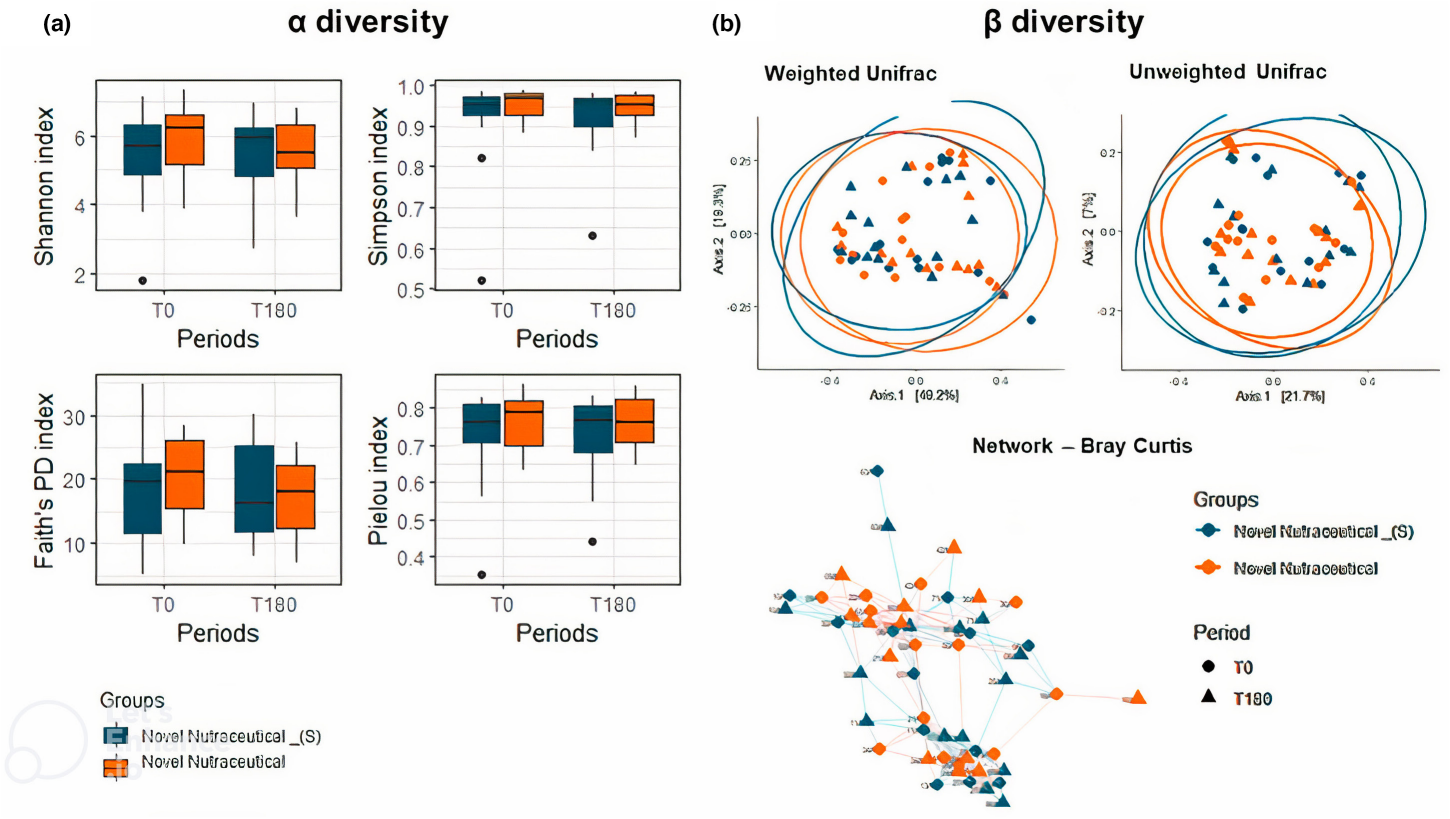
Alpha (α) and beta (β) diversity indices in overweight volunteers at baseline (T0) and 180 days post‐supplementation (T180) with Novel Nutraceutical and Novel Nutraceutical_(S). Boxplot of α‐diversity (a), and (b) β‐diversity. Boxes represent the interquartile range (IQR) between the first and third quartiles (25th and 75th percentiles, respectively), and the horizontal line inside the box defines the median. Whiskers represent the lowest and highest values within 1.5 times the IQR from the first and third quartiles, respectively. “•” indicates >1.5 times and less than three times the IQR.

No statistical difference in microbial composition between groups and periods was observed in terms of alpha (α) diversity indices, including the Shannon index, Simpson index, Faith's PD index, and Pielou index (Figure 3a). Similarly, beta (β) diversity, assessed using Bray–Curtis indices (which consider abundance but not phylogeny) and weighted Unifrac (based on phylogeny), and visualized through the Principal Coordinate Analysis Plot (PCoA) (Figure 3b) did not show differences.

**FIGURE 4:**
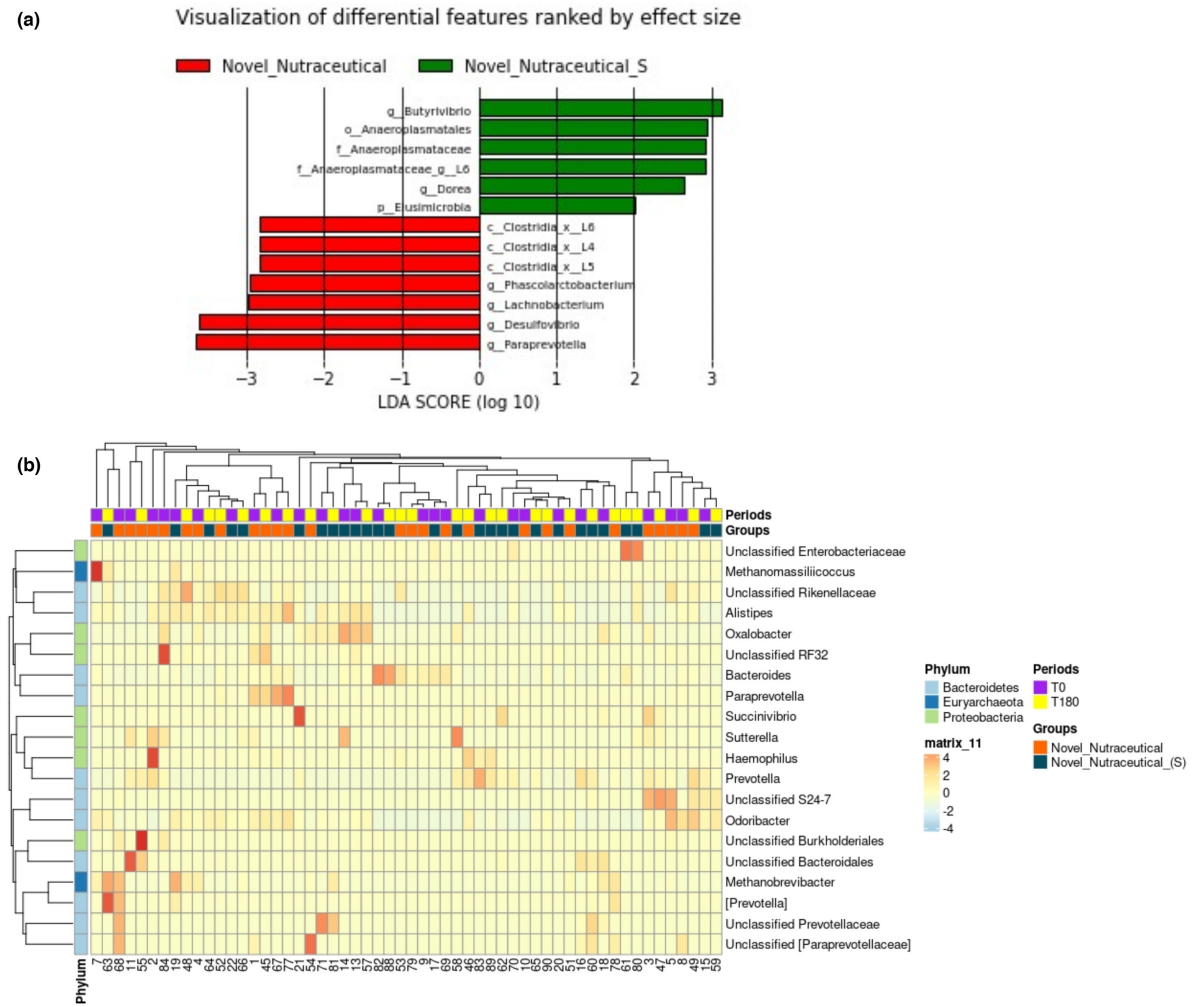
Differentially enriched bacterial taxa in volunteers after 180 days of supplementation by LEfSe (a). Graphical representation of the more abundant bacterial feature after 180 days of supplementation with Novel Nutraceutical_(S), in green, and Novel Nutraceutical, in red. Thirteen differentially abundant taxonomic clades (*α* = 0.05) were identified through the analysis, each possessing an LDA score (log_10_) surpassing 2.0. Heatmap depicting taxonomic readings of volunteers' microbiome (b). The figure highlights variations and groupings in the taxonomic characteristics of volunteers before (T0) in blue and after 180 days of supplementation (T180), in yellow, with Novel Nutraceutical_(S), in purple, and Novel Nutraceutical, in brown. Each column represents the relative abundance, delineated by intensity profiles for individual samples. Colors on the map reveal the relative positioning of read count data, ranging from white to orange signifies values above the mean. The color tones denote the distance of each data point from the mean line. At the sidebar of the heatmap, there is the overall relative abundance of the taxa at a given taxonomic level, represented by three phyla, Bacteroidetes, Euryarchaeota, and Proteobacteria.

The LEfSe analysis was employed to discern features significantly and biologically distinct between the groups of interest, categorizing them based on their effect size. A comparative examination of microbiome data at baseline (T0) and after 180 days (T180) revealed that both Novel Nutraceutical_(S) and Novel Nutraceutical supplements modulated 13 distinct bacterial features, each achieving a Linear Discriminant Analysis (LDA) score higher than two. Following 180 days of Novel Nutraceutical_(S) supplementation a noteworthy increase in the genus *Butyrivibrio*, LDA score higher than three, was accompanied by bacterial features from the phylum Eusimicrobia, order Anaeroplasmatales, family Anaeroplasmataceae, and genus *Dorea*, each exhibiting an LDA score higher than two. Conversely, the supplementation with Novel Nutraceutical resulted in a significant elevation of the genus *Paraprevotella* and *Desulfovibrio*, both with LDA scores surpassing three. Additionally, bacterial features from the Class Clostridia, genus *Phascolarctobacterium*, and L*achnobacterium* exhibited LDA scores higher than two (Figure 4a). To enhance the interpretability of microbiome data, we employed a heatmap visualization technique that specifically highlighted genera showing differential representation between the supplement groups (Figure 4b).

**FIGURE 5:**
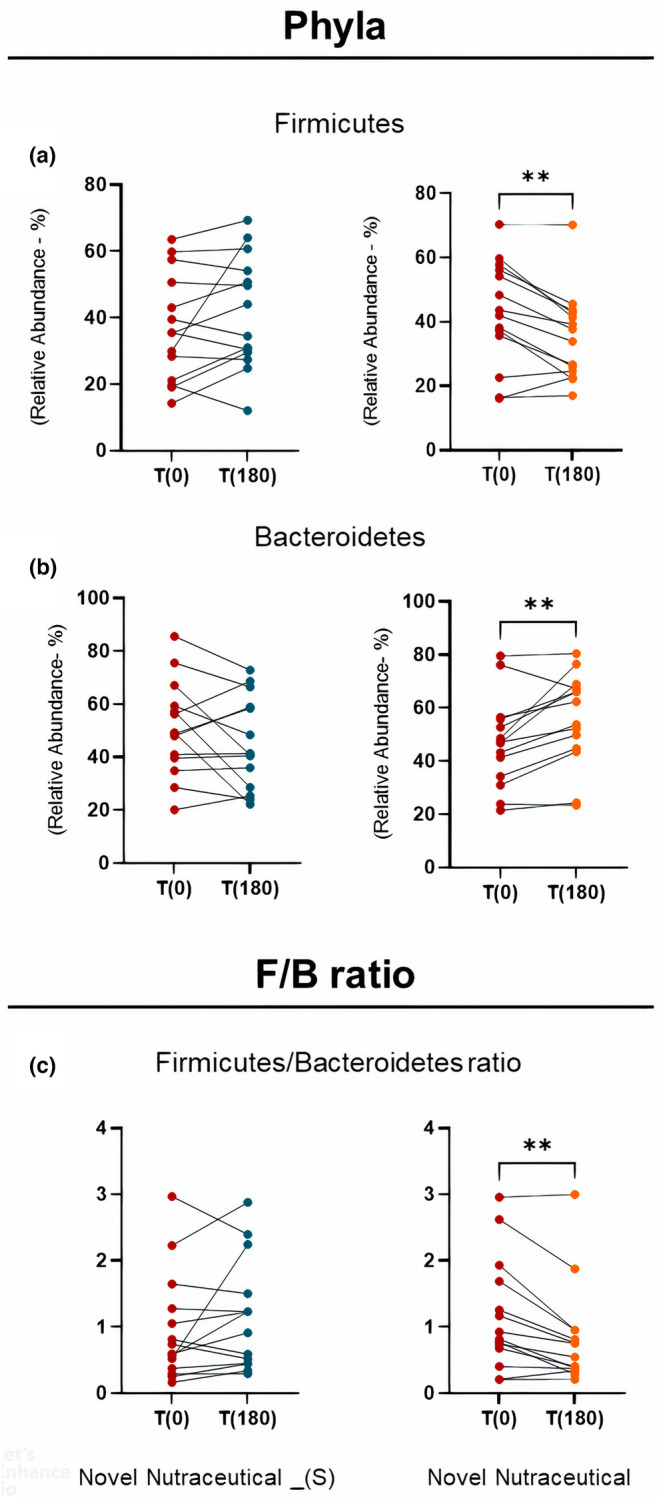
Phyla profiles and F/B ratio from baseline time (T0) and 180 days (T180) post‐supplementation in overweight volunteers' groups taking the different supplements. (a) Relative abundance in % of the Phylum Firmicutes for Novel Nutraceutical_(s) and Novel Nutraceutical; (b) Relative abundance in % of the Phylum Bacteroidetes for Novel Nutraceutical_(S) and Novel Nutraceutical; (c) Ratio – Firmicutes/Bacteroidetes ratio (F/B ratio). Novel Nutraceutical_(S) (*n* = 14) and Novel Nutraceutical (*n* = 14). Values are expressed as the percent of relative abundance (mean ± standard deviation). *p < .05, **p < .01, ***p < .001.

The analysis of 16S rRNA from fecal samples obtained from overweight volunteers administered either Novel Nutraceutical_(S) or Novel Nutraceutical supplements revealed contrasting effects (Figure 5). Comparison of T0 versus T180 data for the phylum Firmicutes (Figure 5a), Bacteroidetes (Figure 5b), and the F/B ratio (Figure 5c) indicated discernible differences. Specifically, Novel Nutraceutical_(S) showed no significant changes in relative abundances when compared to volunteers receiving Novel Nutraceutical after 180 days of supplementation.

### 3.3 The Novel Nutraceutical supplement showed translatability in the gut microbiota between obese mice and overweight humans

While findings from animal models may not always seamlessly extrapolate to humans (Nguyen et al., 2015), our study examined fecal microbiota dynamics in both control and diet‐induced metabolic syndrome mice (Nehmi‐Filho, Santamarina, et al., 2023). Remarkably, our study identified analogous outcomes in taxonomic classes between mice and humans following supplementation with the Novel Nutraceutical.

**FIGURE 6:**
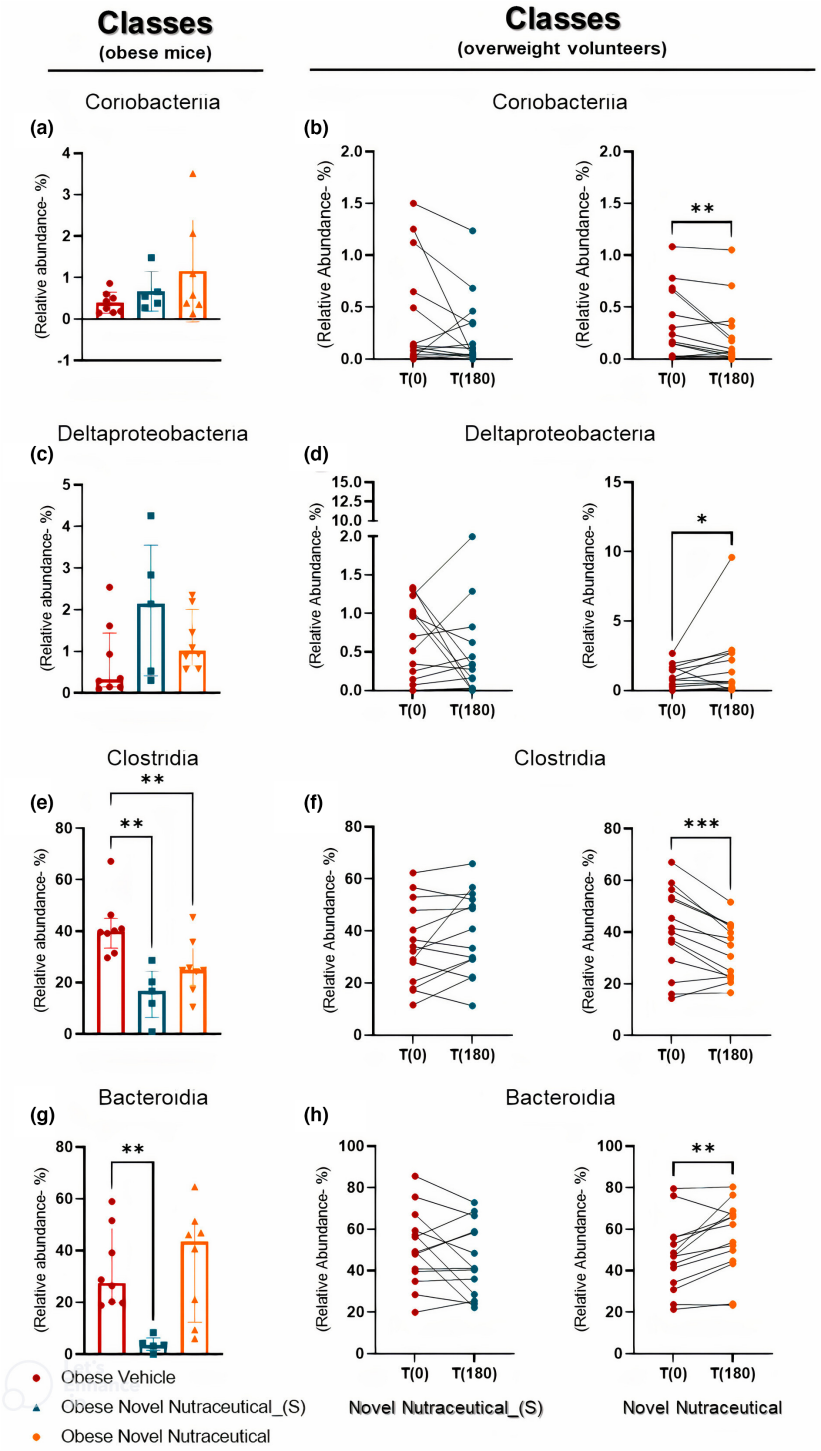
Profile for specific classes comparing results between obese mice and overweight volunteers' microbiome when treated with Novel Nutraceutical_(s) and Novel Nutraceutical. Classes (a) Coriobacteriia (obese mice); (b) Coriobacteriia (overweight people); (c) Deltaproteobacteria (obese mice); (d) Deltaproteobacteria (overweight people at T0 and T180); (e) Clostridia (obese mice); (f) Clostridia (overweight people at T0 and T180); (g) Bacteroidia (obese mice); and (h) Bacteroidia (overweight people at T0 and T180). For overweight people: Novel Nutraceutical_(S) (*n* = 14) and Novel Nutraceutical (*n* = 14). For obese mice, results are derived from 4 to 8 animals. Values are expressed as the percent of relative abundance (mean ± standard deviation). *p < .05, **p < .01, ***p < .001.

During the 28‐day supplementation period in obese‐diet mice groups, receiving oral supplementation with Vehicle, Novel Nutraceutical_(S), and Novel Nutraceutical, we observed no significant differences in the Coriobacteriia (Figure 6a) and Deltaproteobacteria (Figure 6c) classes post‐supplementation. However, in overweight volunteers, the relative abundance of the Coriobacteriia class decreased (Figure 6b), and Deltaproteobacteria increased (Figure 6d) after Novel Nutraceutical supplementation, while remaining unchanged with Novel Nutraceutical_(S) 180 days post‐supplementation compared to the baseline time (T0).

In obese mice groups, all supplementations led to a decrease in the Clostridia class (Figure 6e) in gut microbiota. However, in overweight volunteers (Figure 6f), only the Novel Nutraceutical reduced the relative abundance. Contrasting results were observed in the Bacteroidia class, where mice administered Novel Nutraceutical_(S) (Figure 6g) exhibited decreased relative abundance compared to vehicle‐obese mice. In humans (Figure 6h), the abundance remained unchanged with Novel Nutraceutical_(S), while Novel Nutraceutical supplementation increased relative abundance in overweight volunteers.

**FIGURE 7:**
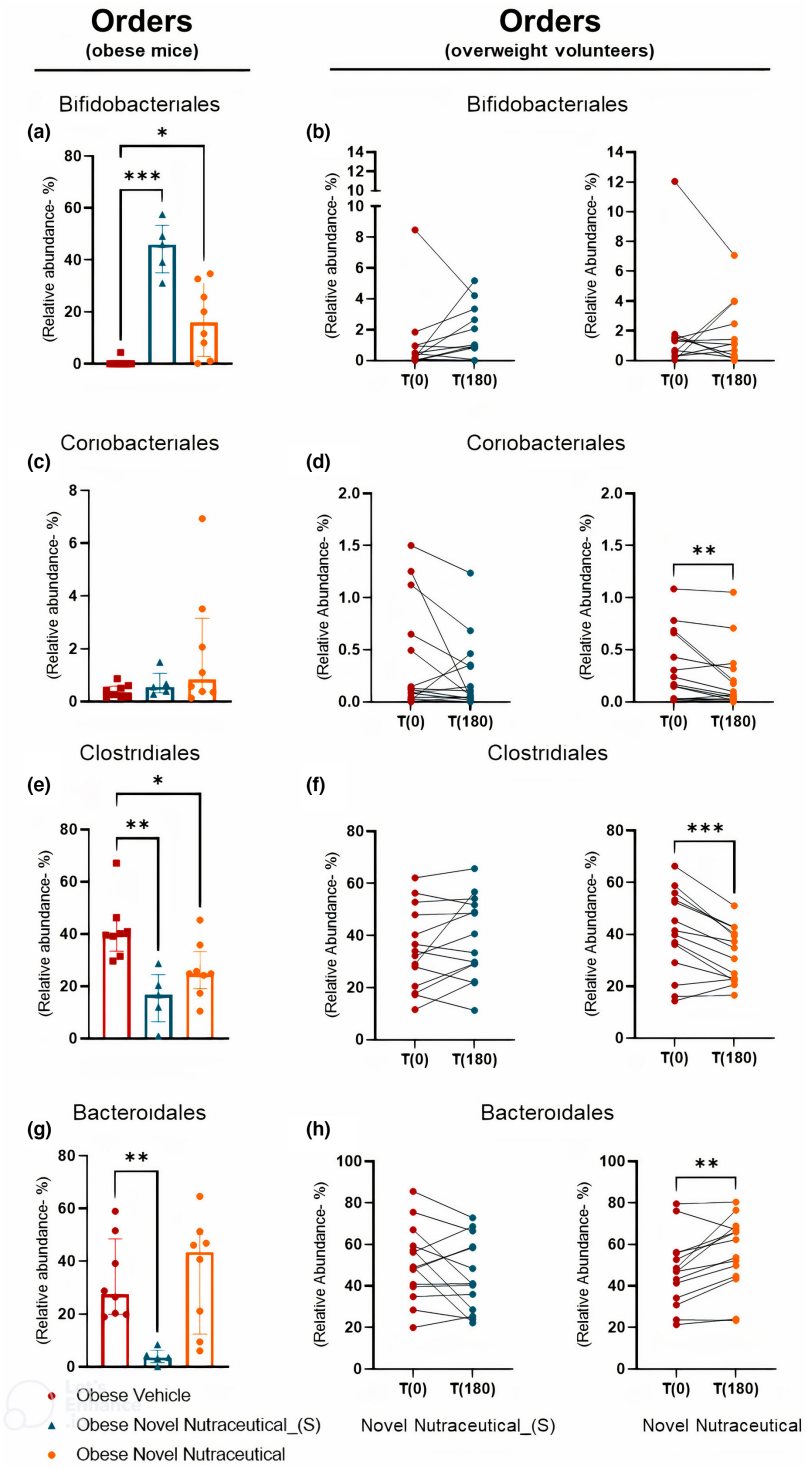
Profile for specific orders comparing results between obese mice and overweight volunteers' microbiome when treated with Novel Nutraceutical_(s) and Novel Nutraceutical. Orders (a) Bifidobacteriales (obese mice); (b) Bifidobacteriales (overweight people at T0 and T180); (c) Coriobacteriales (obese mice); (d) Coriobacteriales (overweight people at T0 and T180); (e) Clostridiales (obese mice); (f) Clostridiales (overweight people at T0 and T180); (g) Bacteroidales (obese mice); and (h) Bacteroidales (overweight people at T0 and T180). For overweight people: Novel Nutraceutical_(S) (*n* = 14) and Novel Nutraceutical (*n* = 14). For obese mice, results are derived from 4 to 8 animals. Values are expressed as the percent of relative abundance (mean ± standard deviation). *p < .05, **p < .01, ***p < .001.

Further exploration at the taxonomy order level uncovered notable distinctions between the effects of Novel Nutraceutical_(S) and Novel Nutraceutical on the gut microbiota of obese mice and overweight volunteers. In obese mice (Figure 7a), both Novel Nutraceutical_(S) and Novel Nutraceutical increased the Bifidobacteriales order, while in overweight volunteers (Figure 7b), no significant alterations in relative abundance were observed. The Coriobacteriales order exhibited negative modulation 180 days after Novel Nutraceutical supplementation in overweight volunteers (Figure 7d). Within the Clostridiales order, both supplementations led to a decreased relative abundance in mice (Figure 7e). However, in overweight volunteers, only Novel Nutraceutical exhibited modulatory effects in this order (Figure 7f). Novel Nutraceutical_(S) decreased the Bacteroidales order in obese mice (Figure 7g), while in overweight volunteers (Figure 7h), only Novel Nutraceutical increased relative abundance in fecal gut microbiota.

### 3.4 The Novel Nutraceutical supplement modulates genera and species within the gut microbiota associated with obesity

**FIGURE 8:**
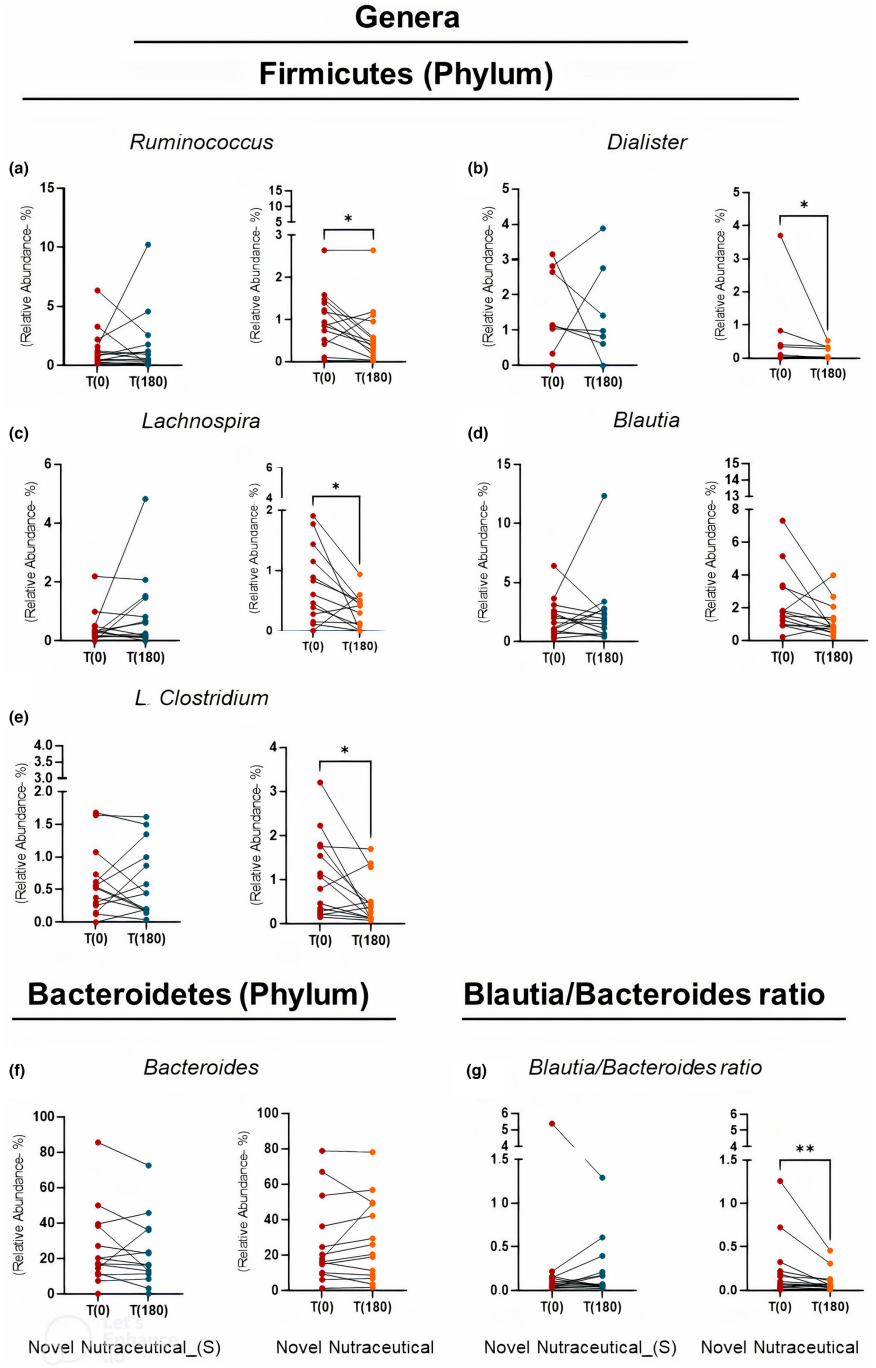
Genera profiles from fecal microbiota at baseline time (T0) and 180 days (T180) post‐supplementation in overweight volunteers' groups taking the different supplements. Genera from Firmicutes phylum (a) *Ruminococcus*; (b) *Dialister*; (c) *Lachnospira*; (d) *Blautia*; (e) *L. clostridium*; Bacteroidetes phylum (f) *Bacteroides*; (g) *Blautia/Bacteroides* ratio. Novel Nutraceutical_(S) (*n* = 14) and Novel Nutraceutical (*n* = 14). Values are expressed as the percent of relative abundance (mean ± standard deviation). *p < .05, **p < .01, ***p < .001.

Among the supplements, only the Novel Nutraceutical exhibited a discernible impact on gut microbiota genera in overweight volunteers. After 180 days of supplementation, significant alterations in relative abundance were observed for specific genera. In the Novel Nutraceutical group, *Ruminococcus* (Figure 8a), *Dialister* (Figure 8b), *Lachnospira* (Figure 8c), and *L. clostridium* (Figure 8e) demonstrated decreased relative abundance, contributing to a shift in the *Blautia/Bacteroides* ratio (Figure 8g). Conversely, Blautia (Figure 8d) and *Bacteroides* (Figure 8f) were not influenced by the Novel Nutraceutical after 180 days of supplementation. These bacterial changes were specifically noted in overweight volunteers who received the Novel Nutraceutical Supplement.

**FIGURE 9:**
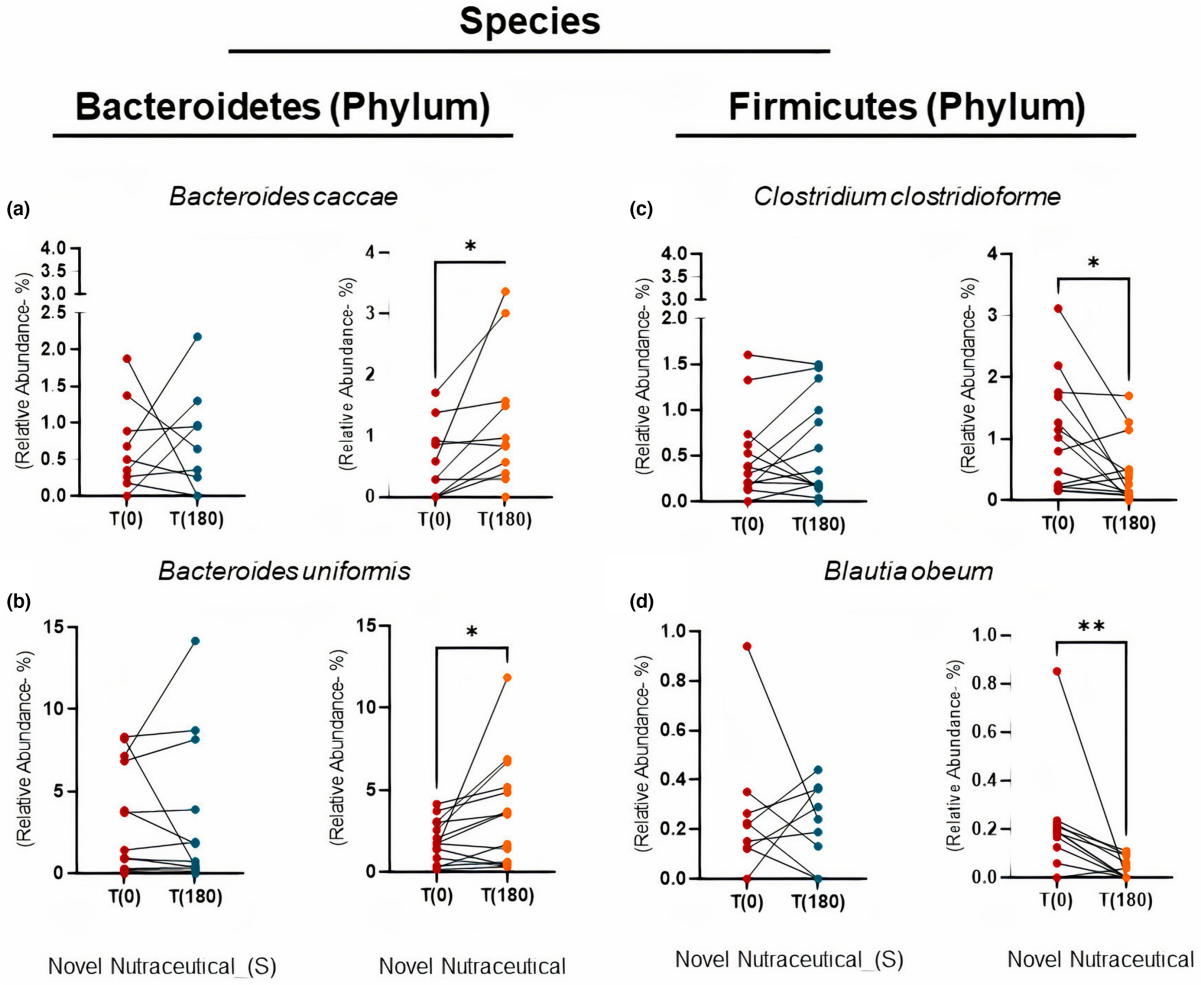
Species profiles from fecal microbiota at baseline time (T0) and 180 days (T180) post‐supplementation in overweight volunteers' groups taking the different supplements. Genus from Firmicutes phylum such as (a) *Bacteroides caccae*; (b) *Bacteroides uniformis*; (c) *Clostridium clostridioforme*; (d) *Blautia obeum*. Novel Nutraceutical_(S) (*n* = 14) and Novel Nutraceutical (*n* = 14). Values are expressed as the percent of relative abundance (mean ± standard deviation). *p < .05, **p < .01, ***p < .001.

Notably, there were absolute increases in the relative abundance of *Bacteroides caccae* (Figure 9a) and *Bacteroides uniformis* (Figure 9b), while *Clostridium clostridioforme* (Figure 9c) and *Blautia obeum* (Figure 9d) showed a decrease in relative abundance at T180 T0. In contrast, the group that received the Novel Nutraceutical (S) did not exhibit changes in the bacterial species from the Bacteroidetes and Firmicutes phyla mentioned earlier (Figure 5).

### 3.5 The Novel Nutraceutical supplement triggers a correlation between predictive markers of hypertension and liver disease with the gut microbiota

The analysis of fecal microbiota in both overweight volunteers and obese mice revealed significant influences of the Novel Nutraceutical on taxonomic profiles and anthropometric measures, such as WHR and weight gain, respectively. The Novel Nutraceutical_(S) (without silymarin) significantly decreased the De‐Ritis ratio at T180 (1.47 ± 0.37) compared to baseline (T0 = 2.47 ± 1.14) (Table 1). Although statistical significance in this ratio was not observed in the group of overweight volunteers receiving the Novel Nutraceutical after 180 days (2.00 ± 0.82) of supplementation, a reduction compared to the baseline (T0 = 2.62 ± 1.18) (Table 1).

To further explore the relationships between the supplements and various parameters, we employed Multiple Linear Regression (MLR) analysis. The goal was to determine whether Novel Nutraceutical_(S) or Novel Nutraceutical supplements could predict shifts in the relative abundance of microorganisms exhibiting significant changes (Phyla, Ratios, Genera, and Species), as well as anthropometric, De‐Ritis ratio, and biochemistry/endocrine parameters after 180 days of supplementation.

The MLR analysis revealed that overweight volunteers receiving the Novel Nutraceutical_(S) showed a weak positive association between Bacteroidetes and the De‐Ritis ratio (AdjCoef 0.368; 95%CI 0.003–0.737: *p* = .048), as well as the *Lachnospira* genus with Body Mass and BMI (AdjCoef 0.005; 95%CI 0.000–0.010: *p* = .042). On the other hand, the Novel Nutraceutical displayed strongly positive associations, including the Firmicutes/Bacteroidetes ratio (AdjCoef 0.850; 95%CI 0.023–1.677: *p* = .045), *Blautia/Bacteroides* ratio (AdjCoef 0.221; 95%CI 0.036–0.406: *p* = .023), *L. clostridium* (AdjCoef 0.488; 95%CI 0.115–0.861: *p* = .015), *Blautia* (AdjCoef 0.258; 95%CI 0.000–0.516: *p* = .050), and *Ruminococcus* (AdjCoef 0.471; 95%CI 0.132–0.809: *p* = .010) genera with the De‐Ritis ratio (Table 3).

**TABLE 3:** Multiple linear regression analysis from gut microbiota with AST/ALT ratio (De Ritis) and anthropometric parameters in supplemented overweight volunteers.

			Coef (IC95% min – IC95% max)	*p*
Novel Nutraceutical_(S)		AST/ALT ratio (De Ritis)		
	Phyla	Bacteroidetes	0.368 (0.003–0.737)	.048
		Body mass		
	Genera	*Lachnospira*	0.005 (0.000–0.010)	.042
		B.M.I.		
	Genera	*Lachnospira*	0.005 (0.000–0.010)	.042
Novel Nutraceutical		AST/ALT ratio (De Ritis)		
	Ratios	Firmicutes/Bacteroidetes	0.850 (0.023–1.677)	.045
		*Blautia*/*Bacteroides*	0.221 (0.036–0.406)	.023
	Genera	*L. clostridium*	0.488 (0.115–0.861)	.015
		*Blautia*	0.258 (0.000–0.516)	.050
		*Ruminococcus*	0.471 (0.132–0.809)	.010
	Species	*Clostridium clostridioforme*	0.437 (0.064–0.811)	.025

Therefore, our data suggest that the observed decrease in the De‐Ritis ratio and anthropometrics measures (Table 1) after 180 days (T180) of Novel Nutraceutical supplementations could be attributed to the corresponding decrease in gut microbiota relative abundance, as depicted in the aforementioned data (Figures 4, 7, 8).

## 4 DISCUSSION

Diet interventions (Nguyen et al., 2015), probiotics (Davis, 2016), fecal bacterial transplantation (Liu et al., 2021), synbiotics, and prebiotics (Davis, 2016) have been explored for their roles in regulating gut microbiota and prevent, improve, or treat overweight or obesity, with its distinct advantages and disadvantages (Marrs & Walter, 2021; Suez et al., 2019). The emergence of nutraceuticals, as non‐pharmacological products, holds promise for comprehensive human health benefits (Ronis et al., 2018). In our research, we investigated a specific nutraceutical composition, the Novel Nutraceutical, incorporating various components (Nehmi‐Filho, Santamarina, et al., 2023). Notably, while existing literature often studies the singular effects of these supplements, our study brings novelty by examining their combined association with yeast β‐glucan, prebiotics, and minerals, with or without silymarin seed extract. The literature available often studies the single effects of these supplements, and thus the association presented here brings novelty. In this study, we aimed to answer the following questions: Can nutraceutical compositions per se induce changes in the gut microbiota, without dietary intervention? If so, could the reshaping of the microbiota be associated with improvements in liver damage biomarkers and/or anthropometric parameters in people with obesity?

Our research demonstrated the significant efficacy of the Novel Nutraceutical in both pre‐clinical and clinical settings. In a pre‐clinical model of diet‐induced metabolic syndrome, the supplement showcased improvements in glycemia, insulin resistance, fibrosis, and fatty liver disease, alongside notable modulation in gut microbiota (Nehmi et al., 2021; Nehmi‐Filho, de Freitas, et al., 2023; Santamarina et al., 2022). Similarly, in a double‐blind randomized clinical trial with overweight volunteers, the Novel Nutraceutical exhibited substantial effects on liver damage biomarkers, endocrine hormones, and various anthropometric parameters (Nehmi‐Filho, Santamarina, et al., 2023).

The analysis of anthropometric parameters revealed a reduction in the WHR over time exclusively in the Novel Nutraceutical group, indicating a potential for body composition remodeling. The reduction in WHR is particularly significant as it serves as a reliable gauge for diagnosing obesity, especially related to visceral adiposity (Nehmi‐Filho, Santamarina, et al., 2023). The supplement's ability to induce alterations in body fat mass distribution without dietary or exercise intervention highlights its potential to enhance insulin sensitivity (Patel & Abate, 2013).

Overweight and obesity pose significant risks for cardiometabolic diseases and nonalcoholic fatty liver disease (NAFLD), a prominent indicator of visceral adiposity. Analyzing serum levels of AST and ALT, along with the AST/ALT ratio (De‐Ritis ratio), stands as an accessible means for clinicians to assess liver damage diagnosing routinely such as steatosis (Maldonado‐Hernández et al., 2017; Nehmi‐Filho, Santamarina, et al., 2023). The results on the De‐Ritis ratio demonstrated a potential hepatoprotective effect against NAFLD and NASH by the supplementation. Enhancing NAFLD management could represent an initial step in the metabolic recuperation from obesity‐related ailments, promoting the restoration of insulin signaling and lipid metabolism. Notably, this aligns with our earlier pre‐clinical research showcasing the anti‐diabetic effects (Nehmi et al., 2021; Santamarina et al., 2022).

Regarding the dietary intake patterns, a slight upsurge in carbohydrate intake was noted among volunteers in the Novel Nutraceutical group over time in the absence of diet counseling. The increase in carbohydrate consumption potentially bears relevance to the profile of the intestinal microbiota, potentially leading to a reduction in microbial diversity favoring the proliferation of adverse bacterial colonies (Kawano et al., 2022). Although dietary choices significantly influence gut microbiota composition (Rasmussen et al., 2009), our findings unequivocally underscore the direct and exclusive impact of the nutraceutical compositions on microbiota. It is critical to emphasize that despite the heightened carbohydrate intake observed in the Novel Nutraceutical group at T180, the supplement retained its efficacy in promoting the reduction of visceral adiposity and fostering favorable remodeling of the gut microbiota.

The gut microbiota's pivotal role in the onset of metabolic diseases linked to overweight and obesity has garnered significant recognition (Gomes et al., 2018). Nonetheless, the microbiome profile of people grappling with overweight and obesity was previously depicted as sharing a similar bacterial phenotype (Crovesy et al., 2020; León Aguilera et al., 2022). While gut microbiota diversity and richness remained unchanged during the supplementation period, specific modulations were observed, likely attributed to the synergistic effects of nutraceutical components. The inclusion of silymarin in the Novel Nutraceutical composition played a prominent role in these alterations. The study highlighted the potential benefits of prebiotics, such as β‐glucans, in regulating carbohydrate and lipid metabolism (Cronin et al., 2021).

The Novel Nutraceutical, particularly with silymarin, exhibited the capacity to modulate the relative abundance of the Bacteroidetes phylum, challenging the conventional notion of a shared bacterial phenotype in overweight and obese volunteers (Nehmi‐Filho, de Freitas, et al., 2023). The supplement positively influenced specific species associated with normal weight and weight loss, indicating its potential in metabolic and endocrine disorder intervention (Bischoff et al., 2022; Del Chierico et al., 2018). Silymarin has exhibited anti‐diabetic and anti‐obesity properties, as demonstrated by Nehmi et al. (2021). However, Valentová et al. (2020) revealed that silymarin's biotransformation, particularly at higher doses (pharmacological/200 g/L), tends to display resistance to microbial breakdown in the gut. Conversely, at concentrations lower than 10 mg/L, complete degradation occurred within 16 hours in ex vivo models. This variance in resistance or degradation hinges significantly upon the distinct structures of these isomeric compounds, known as flavonolignans, and also on the individual characteristics of stool donors. The microbiota exhibits wide‐ranging diversity and individual patterns, influencing the interaction between microorganisms and host physiology (Makki et al., 2018).

Prebiotics, categorized as non‐digestible substances, can modulate the microbiome (Gong & Miao, 2019). They regulate blood glucose and serum lipid levels by reducing their absorption at the intestinal level. Notably, β‐glucans possess bifidogenic properties, thereby aiding in regulating carbohydrate and lipid metabolism (Cronin et al., 2021). Nonetheless, excessive intake of prebiotics like fructooligosaccharides (FOS) and galactooligosaccharides (GOS) can evoke contrasting effects, leading to increased glycemia and alterations in microbiota composition (Owen et al., 2017; Santamarina et al., 2022).

Prior studies have highlighted the benefits of protocols involving prebiotics such as FOS (Everard et al., 2013), GOS (Lakshmanan et al., 2021), β‐glucans (Arena et al., 2017), and mineral supplementation comprising magnesium (Santos‐Marcos et al., 2019), zinc (Shen et al., 2019), and selenium (Kumar et al., 2020), alongside plant‐derived compounds like flavonolignans (silymarin) (Xu et al., 2022). These protocols have exhibited advantages in ameliorating the pathogenesis of various noncommunicable chronic illnesses, such as obesity and cardiovascular diseases. The synergistic effect resulting from the combination of these compounds, known as a symbiotic effect offers an enhanced efficacy that surpasses individual effects. As a result, they hold promise as valuable tools for non‐pharmacological intervention in obesity‐related metabolic and endocrine disorders (Mahlapuu et al., 2016; Nehmi et al., 2021; Santamarina et al., 2023).

Dysbiosis in overweight and obese individuals is often linked to a decrease in the anti‐obesogenic Bacteroidetes phylum, an increase in the obesogenic Firmicutes phylum, and heightened levels of the genus *Clostridium* (Davis, 2016; Gomes et al., 2018). Ratios such as Firmicutes/Bacteroidetes and Blautia/Bacteroides have also been associated with obesity (Kim et al., 2022; Palmas et al., 2021; Stojanov et al., 2020). Nonetheless, recent findings present contrasting views, indicating no significant differences in these ratios and Bacteroidetes abundance between obese and lean individuals (Cheng et al., 2022). Our study revealed that the Novel Nutraceutical supplement exerted a positive influence on the Bacteroidetes phylum, particularly by augmenting the abundance of *Bacteroides caccae*. This species has been associated with normal weight in adolescents (Del Chierico et al., 2018) and is considered a predictor of weight loss (Bischoff et al., 2022). Furthermore, the supplement increased the abundance of *Bacteroides uniformis*, contributing to elevated gut folate levels, regulating liver lipid metabolism (Roelofs et al., 2016), and constituting a part of the gut microbiota (Lee et al., 2021). Consistently, in our obese mice model, the supplement similarly elevated the abundance of the Bacteroidaceae family and the *Bacteroides* genus (Nehmi‐Filho, de Freitas, et al., 2023).

Notably, the Clostridia gut population has been associated with dysmetabolism and dysbiosis, escalating in high‐fat diets (León Aguilera et al., 2022), in individuals with obesity (Gomes et al., 2018). However, the *Clostridium* genus within the Clostridia class is a butyrate‐producing bacterium that exhibits anti‐inflammatory properties (Kim et al., 2020; León Aguilera et al., 2022). Intricate mechanisms linked to fatty acid metabolism potentially connect Firmicutes and Clostridium clusters with obesity (Nadal et al., 2009). *Blautia*, a genus within the Bacteroidetes phylum, has been implicated in exacerbating hepatic inflammation by heightening intestinal permeability among overweight and obese individuals. This exacerbation arises from its production of lipopolysaccharides (LPS), which trigger a pro‐inflammatory response, contribute to obesity, and facilitate the progression of fatty liver disease (Liu et al., 2022).

*Blautia obeum* and *Clostridium clostridioforme* are observed to increase the gut microbiota of young and middle‐aged adults with prediabetes, echoing our findings in sedentary overweight volunteers before supplementation. This rise in abundance was linked to a diminished microbial capacity to metabolize dietary polyphenols (Zhang et al., 2020). Interestingly, the group receiving the Novel Nutraceutical displayed alterations in gut microbiome composition, a change not observed in the Novel Nutraceutical_(S) group during the supplementation period. Furthermore, here we noted positive correlations between these microbiota shifts (including Bl*autia/Bacteroides* and Firmicutes/Bacteroidetes ratios) and the De‐Ritis ratio.

Our findings suggested that the Novel Nutraceutical exhibited a predictive capacity in reshaping the microbiota, leading to enhancements in clinical parameters such as WHR and the De‐Ritis ratio (Nehmi‐Filho, Santamarina, et al., 2023). These outcomes imply that microbiota modulation is a primary mechanism of action for the Novel Nutraceutical in improving metabolic parameters and contributing to hepatic function recovery.

Despite limitations, such as sample size and gender distribution, our study suggests that the 180‐day post‐supplementation administration of the Novel Nutraceutical effectively altered intestinal microbiota composition and an important liver damage biomarker in sedentary overweight Brazilian volunteers, independent of exercise or diet (Nehmi‐Filho, Santamarina, et al., 2023). These findings underscore the translational impact of supplements, bridging the gap between scientific research and clinical practice, and making nutraceuticals more pragmatic and applicable for medical practitioners.

## AUTHOR CONTRIBUTIONS

**Victor Nehmi‐Filho:** Conceptualization (equal); resources (equal); visualization (equal). **Jessica Alves de Freitas:** Formal analysis (equal); methodology (equal); validation (equal). **Lucas Augusto Franco:** Formal analysis (equal); investigation (equal); methodology (equal); writing – review and editing (equal). **Roberta Cristina Martins:** Data curation (equal); formal analysis (equal); investigation (equal); methodology (equal); writing – original draft (equal). **José Antônio Orellana Turri:** Formal analysis (equal); validation (equal). **Aline Boveto Santamarina:** Validation (equal); writing – review and editing (equal). **Joyce Vanessa da Silva Fonseca:** Methodology (equal). **Ester Cerdeira Sabino:** Methodology (equal). **Bruna Carvalho Moraes:** Formal analysis (equal); methodology (equal); writing – original draft (equal). **Erica Souza:** Visualization (equal). **Gilson Masahiro Murata:** Methodology (equal). **Silvia Figueiredo Costa:** Visualization (equal). **Paulo Sérgio Alcântara:** Visualization (equal). **José Pinhata Otoch:** Visualization (equal). **Ana Flávia Marçal Pessoa:** Conceptualization (equal); formal analysis (equal); investigation (equal); methodology (equal); project administration (equal); supervision (equal); validation (equal); visualization (equal); writing – original draft (equal); writing – review and editing (equal).

## FUNDING INFORMATION

This study was financed by Efeom Nutrition S.A., (01/04–21), ECS e LAMF was supported by FAPESP 18/14389‐0 (http://caddecentre.org/), Rede Corona omica BR MCTI/FINEP affiliated to RedeVirus/MCTI (FINEP 01.20.0029.000462/20, CNPq 404096/2020‐4), Bill & Melinda Gates Foundation (INV‐034540), and CAPES (88887.703169/2022‐00).

## CONFLICT OF INTEREST STATEMENT

The authors Nehmi‐Filho, V., and Otoch, J.P making part of Efeom Nutrition S.A that may in any way gain or lose financially from the publication of the manuscript, either now or in the future. The authors Freitas, J.A and Pessoa, A.F.M. received a salary from Efeom Nutrition S.A. The authors Nehmi‐Filho, V., Otoch, J.P, and Pessoa, A.F.M hold patents relating to the content of the manuscript. The authors Franco, L.A.M, Fonseca, J.V.S; Martins, R.C.R; M. Murata, M.G; Costa, S.F, Sabino, E.C; Souza, E; Santamarina, A.B, Alcântara, P.M.D, Turri, J.A.O and Costa, S.F have no competing interests.

## ETHICS STATEMENT

The animal procedure was approved by the Research Ethics Committee of the University of São Paulo School of Medicine, Sao Paulo, Brazil (numbers: 1185/2018 and 1519/2020). All experiments were conducted in accordance with the National Institutes of Health guidelines and the study is reported in accordance with ARRIVE guidelines. The clinical trial was approved by the Ethics Committee for the Analysis of Research Projects (CAPPesq) according to the Declaration of Helsinki under the CAAE number 39984320.5.0000.0068 and registered under identification number NCT04810572 (ClinicalTrials.gov). Furthermore, this study received approval from the Brazilian National System of Genetic Registration (SisGen) under the number AC29D69.

## PATENTS

The formulation of the supplement (patent number: BR 102020 016,156 3) can be found at Revista de Propriedade Industrial n° 2667, accessed on 15 February 2022.

## Data Availability

The datasets generated and/or analyzed during the current study are available in the GenBank® repository, Bioproject PRJNA941000. Link: https://www.ncbi.nlm.nih.gov/sra/PRJNA941000 (Release date: 2024‐09‐30). The data that support the findings of this study are available on request from the corresponding author. This link is exclusive for the reviewers: https://dataview.ncbi.nlm.nih.gov/object/PRJNA941000?reviewer=lfevhd0p2jn9358imrei528946.
